# Supplementary material for: Effect of retinoic acid treatment on the retinoic acid signaling pathway in a human siRNA-based aniridia limbal epithelial cell model, in vitro
Source: PLoS One. 2025 Jun 18;20(6):e0324946. doi: 10.1371/journal.pone.0324946 (PMC12176239; doi:10.1371/journal.pone.0324946)
Supplement: S1 Fig — CYP26A1 (a) and RARA (b) western blot show low protein expression levels in limbal epithelial cells (LECs), compared to positive control Hep G2 and MCF 7 for CYP26A1 and MCF7 for RARA. (DOCX) [file pone.0324946.s001.docx]

**Effect of retinoic acid treatment on the retinoic acid signaling pathway in the siRNA-based aniridia limbal epithelial cell model, *in vitro***

Shao-Lun Hsu ^1^, Tanja Stachon ^1^, Fabian N. Fries ^1, 2^, Zhen Li ^1^, Shuailin Li^1^, Shanhe Liu^1^, Berthold Seitz ^2^, Swarnali Kundu^1^, Maryam Amini ^1^, Shweta Suiwal ^1^, Nóra Szentmáry ^1^

^1^ Dr. Rolf M. Schwiete Center for Limbal Stem Cell and Congenital Aniridia Research, Saarland University, Homburg/Saar, Germany

^2^ Department of Ophthalmology, Saarland University Medical Center, Homburg/Saar, Germany

| 1. CYP26A1 representative western blot | 1. RARA representative western blot |
| --- | --- |
| 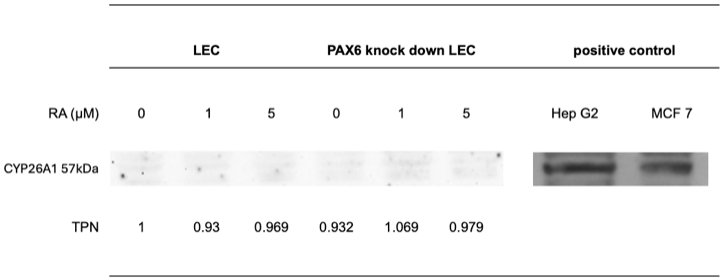 | 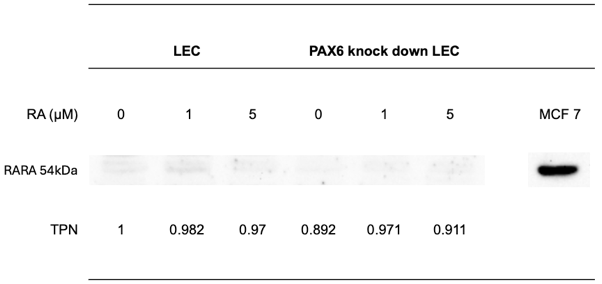 |

**Figure 1. CYP26A1 and RARA Western blot analyzis.** CYP26A1 (a) and RARA (b) western blot show low protein expression levels in limbal epithelial cells (LECs), compared to positive control Hep G2 and MCF 7 for CYP26A1 and MCF7 for RARA.
