# Supplementary material for: Effect of retinoic acid treatment on the retinoic acid signaling pathway in a human siRNA-based aniridia limbal epithelial cell model, in vitro
Source: PLoS One. 2025 Jun 18;20(6):e0324946. doi: 10.1371/journal.pone.0324946 (PMC12176239; doi:10.1371/journal.pone.0324946)

Fig. 2c (PAX6)

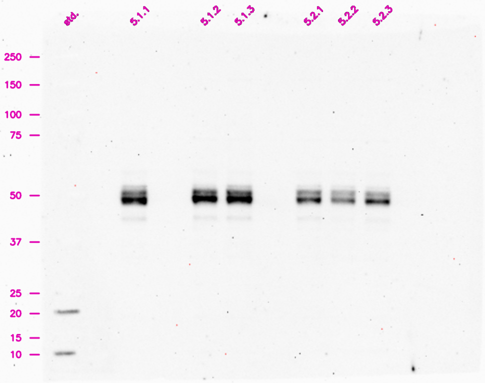

Fig. 3c (RDH10)

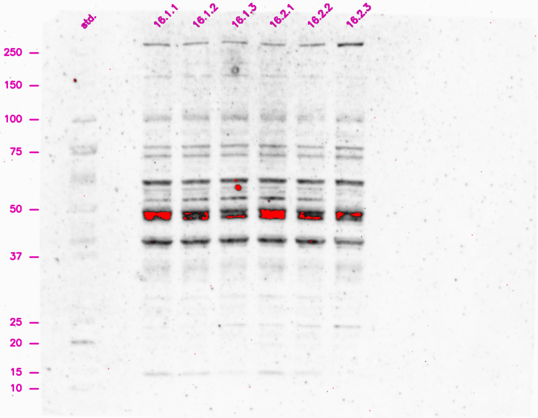
std.
16.1.1
16.1.2
16.1.3
16.2.1
16.2.2
16.2.3
250
150
100
75
50
37
25
20
15
10

Fig. 3f (ADH7)

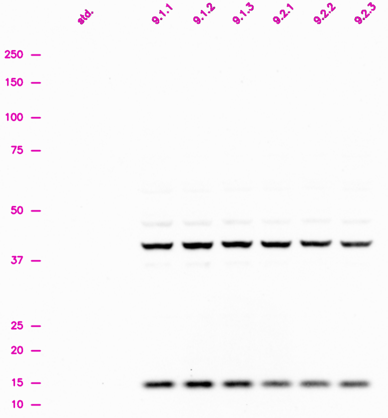

Fig. 3i (ALDH1A1)

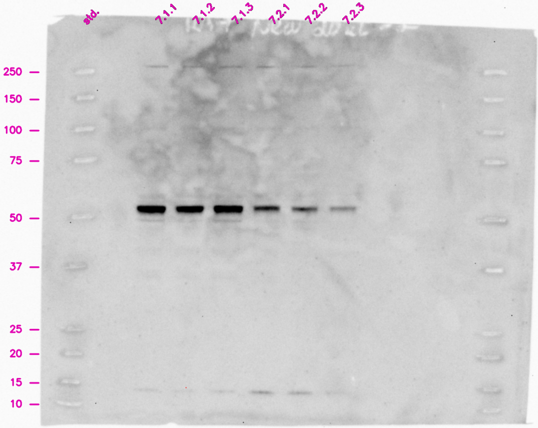

Fig. 4c (CRABP2)

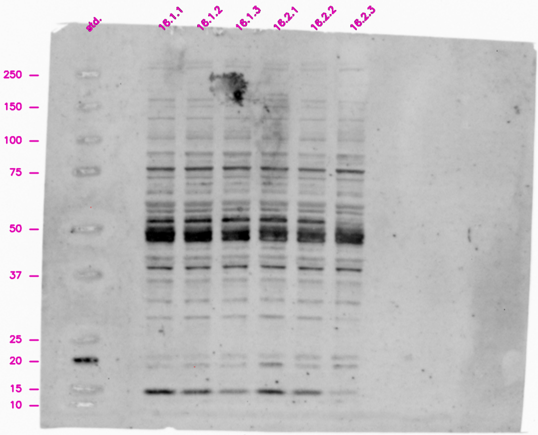

Fig. 4f (FABP5)

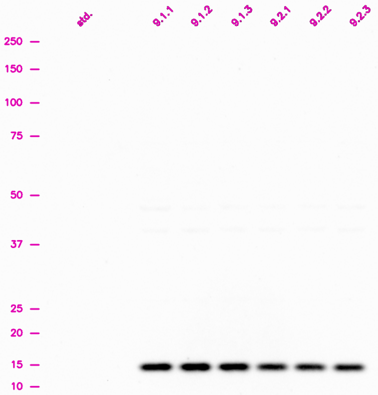

Fig. 5c (PPARG2)

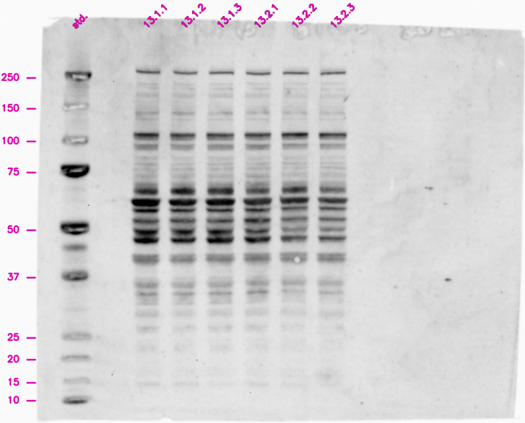

Fig. 5f (RXRA)

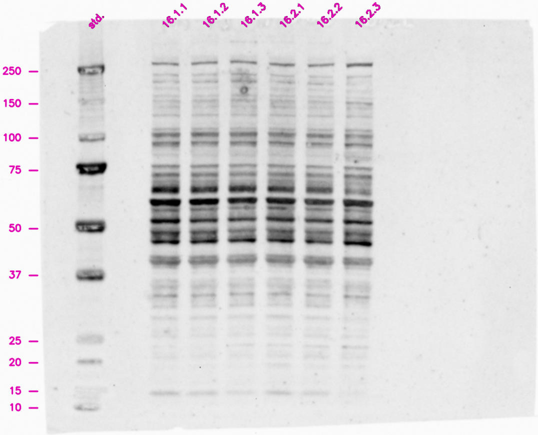

std.
16.1.1
16.1.2
16.1.3
16.2.1
16.2.2
16.2.3
250
150
100
75
50
37
25
20
15
10

Fig. 5k (RARB)

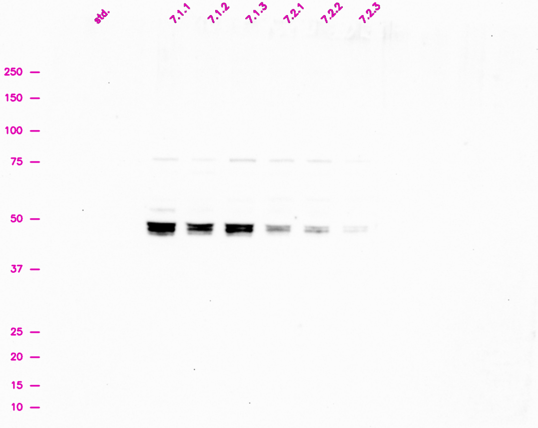

S1 Fig. a (CYP26A1)

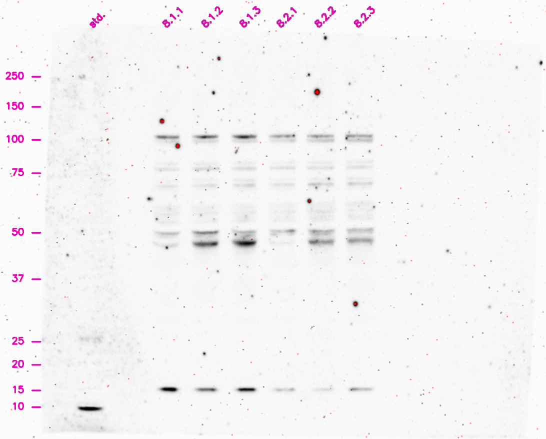

S1 Fig. a (positive control)

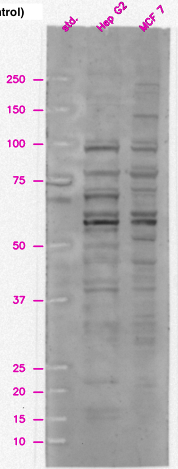

S1 Fig. b (RARA)

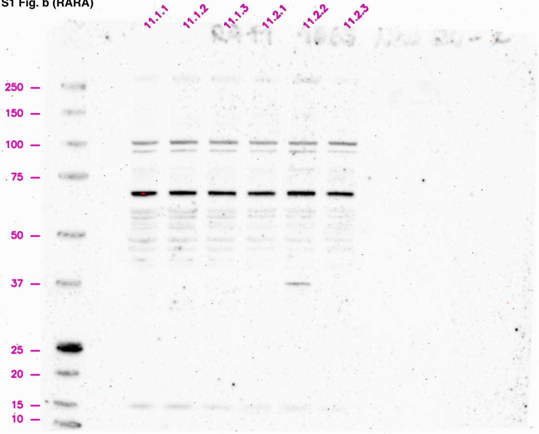

S1 Fig. b (positive control)

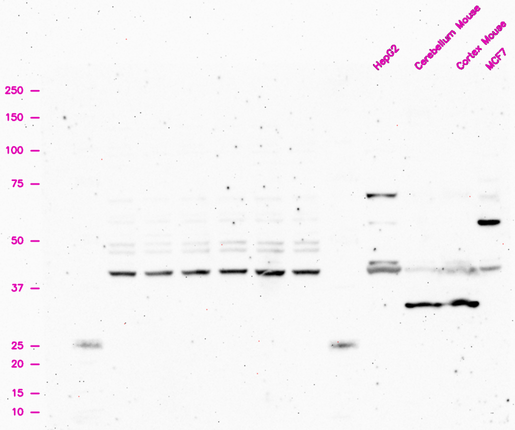

Supplement: S1 File — (PDF) [file pone.0324946.s002.pdf]
